# Analyzing the Virchow pioneering report on brain corpora amylacea: shedding light on recurrent controversies

**DOI:** 10.1007/s00429-023-02664-5

**Published:** 2023-06-26

**Authors:** Marta Riba, Clara Romera, Raquel Alsina, Gerard Alsina-Scheer, Carme Pelegrí, Jordi Vilaplana, Jaume del Valle

**Affiliations:** 1 Secció de Fisiologia, Departament de Bioquímica i Fisiologia, Facultat de Farmàcia i Ciències de l’Alimentació, Universitat de Barcelona, 08028 Barcelona, Spain; 2 Institut de Neurociències, Universitat de Barcelona, 08035 Barcelona, Spain; 3 Centros de Biomedicina en Red de Enfermedades Neurodegenerativas (CIBERNED), 28031 Madrid, Spain; 4 Barcelona, Spain

**Keywords:** Corpora amylacea, Wasteosomes, Brain, Neoepitopes, Glymphatic system, Chronic glymphatic insufficiency

## Abstract

The first report of corpora amylacea (CA) is attributed to Morgagni, who described them in the prostate in the eighteenth century. Nearly a hundred years later, and following the lead started by Purkinje, Virchow described them in the brain. He made a detailed description of the most useful techniques to visualize them, but he failed to describe the cause of why CA do appear, why they are mainly linked with the elderly, and which is their clinical significance. Although in the last two centuries CA have received little attention, recent data have been able to describe that CA accumulate waste products and that some of them can be found in the cerebrospinal fluid and lymphatic nodes, after being released from the brain. Indeed, CA have been renamed to wasteosomes to underline the waste products they gather and to avoid confusion with the term amyloid used by Virchow, now widely related to certain protein deposits found in the brain. Here, after providing a commented English translation of Virchow’s findings, we provide a recent update on these structures and their connection with the glymphatic system insufficiency, for which wasteosomes should be considered a hallmark, and how these bodies could serve as diagnostic or prognostic markers of various brain conditions.

## Introduction

The first reports of corpora amylacea (CA) in the human body have been attributed to Morgagni (Morgagni 1779), who described them in the human prostate. Although Purkinje reported in a conference the presence of these bodies in the central nervous system (Purkinje 1839), it is Rudolf Virchow who, in a scientific article written in German, was the first to accurately describe CA in the brain (Virchow 1854). In the same paper, he described the methods that he had used to characterize these aggregates and the similarities and differences that CA expressed with starch-like substances of vegetal origin. Throughout the second half of the nineteenth century, several authors investigated the origin or the function of Virchow’s recently detailed CA, and considered them a product derived from glial origin, but others also described them as derived from the blood or even the lymphatic circulation, reviewed in (Catola and Achúcarro 1906). Within the first decades of the twentieth century, Buzzard and Greenfield did not consider CA to have any pathological significance (Buzzard and Greenfield 1923), and ten years later Ferraro and Damon wrote an article reviewing what had been published on the origin of these bodies in tissues and describing these aggregates as ‘post mortem artefacts’ formed by protein precipitates (Ferraro and Damon 1931). Then, although some reports about CA were published, the interest in these bodies declined in the following decades (Riba et al. 2021b).

Several decades after the decline in CA interest and more than 100 years after their first reports in the brain, research was resumed and different authors suggested them as structures that accumulate waste products from neurons and different glial cells (Singhrao et al. 1994; Sbarbati et al. 1996) during aging and several diseases (Cavanagh 1999), and their structure was described as based on glucose-containing polymers (Sakai et al. 1969), confirming Virchow’s observations regarding their similarity to cellulose.

More recent studies from the second decade of the 21th century described that CA are made up of polyglucose aggregates, are formed inside astrocytes, express neo-epitopes (NE) and amass waste products present in the brain (Augé et al. 2019; Riba et al. 2021b). Furthermore, these bodies have been reported to leave the brain to the cerebrospinal fluid (CSF), and from this fluid they reach the cervical lymph nodes to be phagocytosed by macrophages (Riba et al. 2019, 2021a). Provided the fact that the term “*amylacea”* in CA refers to vegetal starch-derived substances as already described by Virchow in (Virchow 1854), and to avoid confusion with the family of amyloid peptides associated with Alzheimer’s disease (AD) and other neurodegenerative diseases, our group named these substances as “wasteosomes” focusing on their physiological function as waste containing bodies related to brain cleaning functions (Riba et al. 2021b).

Here, we provide an English translation of the first known article about CA in the nervous system, published by Virchow (Virchow 1854), and a posterior comment discussing the latest results and perspectives of CA compared with the findings that he made and all the opened questions that were left unanswered in 1854.

## Translation

On a substance found in the human brain and spinal cord with the chemical reaction of cellulose. By Rudolf Virchow.

As it is well-known, Carl Schmidt was the first to discover cellulose in ascidia, a component of animal tissue that was previously only known in plants, and the investigations of von Kölliker and Löwig, von Schacht, and von Huxley confirmed this important experience. However, it was always limited to a comparatively low class of invertebrates. The further discovery that Gottlieb made in *Euglena viridis*, namely, that the Infusorium contains paramylon, a body isomeric to cornstarch, was even more relevant for a creature from the lowest classes of the animal kingdom. In vertebrates, on the other hand, nothing similar was known, and only the discovery of Cl. Bernard, that the liver produces sugar, was able to remind us that the substances of the starch family also want to be represented.

From a histological point of view, it struck me that the human umbilical cord has a high structural resemblance to the cellulose tissue of the ascidia (Würzb. Verh. 1851. Vol. II. S, note 161) and I was only strengthened in this view even more by the communications by von Schacht, so I have carefully focused my investigations on this subject. This was frequently in vain, such as in the eggs of amphibians and fish, where I described strange yolk plates (Zeitschr. f. wiss. Zoologie. 1852. Vol. IV. p. 240.).

I felt happier when I recently focused my attention on the so-called corpora amylacea in the brain, I had not had a precise judgment on their exact nature in comparison to the other human amyloid bodies (Würzb. Verh. 1851. Vol. II. p. 5’I.). It has now been found that these take on a pale blue hue after iodine is added and afterward when sulfuric acid is added, they show the beautiful violet color known from cellulose, which is more intense here, because it contrasts sharply with the yellow or brown-colored nitrogenous surrounding substance.

I have repeated this procedure so many times, and with so many precautions, that I consider the result to be absolutely certain. Not only did I perform comparative examinations on several human cadavers at various regions, but I also allowed the agents to act under all possible conditions. The most appropriate procedure here is the one used by Mulder and Harting for plant cellulose (see Moleschott Physiologie des Stoffwechsels S. 103.), by first using an aqueous iodine solution and then hydration with sulfuric acid. The iodine solution must not be too strong, otherwise disturbing iodine precipitates can be obtained, but one must take extra care that the iodine has had a proper effect on the substance. Given the volatility of the substance and the slight attraction to animal substances, this usually happens very unevenly. The edge of the object may only be penetrated, but not the center. Even in places close to each other, one spot receives iodine, and another one does not. It is therefore always advisable to repeat the application of the iodine several times, but without applying too much of it. Adding sulfuric acid obtains a very dark, red–brown color is obtained if the effect is too strong. The safest way is to let sulfuric acid act very slowly. Indeed, I got the most beautiful objects when I left a preparation for 12–24 h covered with the cover glass and in contact with a drop of sulfuric acid at the edge of the cover glass. Then, sometimes, the most beautiful bright violet–blue appeared. Finally, I must mention that random admixtures of starch or cellulose can take place very easily, especially from the cloths with which the object and cover glasses are cleaned, and very easily barrels or smaller scales remain, which later give the reaction.

If one takes care of all of this, the results are as follows.

1. Corpora amylacea (Purkinje) are chemically different from the concentric-spherical bodies that make up the brain sand (*gehirnsand*)^1^, and with which they have mostly been confounded until now. The organic basis of the brain sand particles is apparently nitrogenous: they become intensely yellow due to iodine and sulfuric acid. This applies not only to the sand of the pineal gland (*zirbeldrüse*)^2^ and the vein plexus (*choroid* *plexus*)^3^, but also to that of the Pacchionian (*arachnoid*)^4^ granulations and dura mater, and finally to the platelets of the spinal arachnoid. In all these parts I have never obtained the blue reaction anywhere except in a couple of places in the pineal gland. It should therefore be useful in the future to restrict the name of the Corpuscula amylacea to the cellulose bodies.
2. The cellulose bodies, as I have found so far, are only in the substance of the ependyma ventriculorum and its prolongations. In particular, I include the coating of the cerebral ventricles and the translucent mass in the spinal cord described by Kölliker (Mikrosk. Anat. Bd. II. 1. 413.) as the substantia grisea centralis. Regarding the brain ventricles, I have repeatedly stated that I find them covered entirely by a skin that can be considered among the tissues of the connective substance, on which the epithelium sits. This skin, in turn, contains very fine cellular elements in its internal side and a sometimes denser, sometimes softer basic substance, and it continues inwards without special boundaries between the nerve elements. In the deeper layers of this skin, in the vicinity of the nerve fibers, the cellulose bodies are found most frequently, and again especially abundantly where the ependyma is very thick. Thus, are particularly abundant at the septum, fornix, stria cornea, and in the 4th ventricle. In the spinal cord, the substance corresponding to ependyma lies in the middle of the gray matter, at the point where the spinal canal runs in the fetus. It represents a rudiment of the obliterated canal similar to that seen in the so frequent obliteration of the posterior horn of the lateral ventricles. On cross sections, it is easily recognized as a jelly-like, somewhat resistant mass, which is very easy to isolate. Their cells are much larger and more complete than those of the cerebral ependyma; since they are sufficiently known from Kölliker, I can easily refrain from their description. This spinal ependyma forms a continuous, jelly-like filament up to the filum terminale and may therefore be most appropriately described as the central ependymal filament. The cellulose bodies are also found in it, but, as it seems, more frequently in the upper part than in the lower. On the other hand, I have searched for them in other places in vain so far, in particular, I was not able to find them anywhere on the outer cortex or within the interior of the brain substance.
3. Since it was obvious after the experiment of Cl. Bernard, who produced sugar urine in rabbits by injuring the floor of the 4th ventricle, to associate the cellulose with it, I also investigated in rabbits, but in vain. I only found a very nice pavement epithelium with very long ciliated cilia, but no cellulose in the fourth, third and lateral ventricles.
4. Hence, the cellulose bodies, therefore, appear to be bound everywhere with the presence of a certain thickness of ependymal substance, and can probably be regarded as a part of the ependyma. However, it was not possible for me to see how they emerge from it. They occur extremely small, so that they hardly correspond to the nuclei of the ependyma. Should they be able to form from these? The larger they become, the more clearly they appear to be layered. Nowhere on them, however, a nitrogenous admixture is visible, recognizable by yellow staining. Only the center tends to be darker blue, i.e., denser, than the marginal layers.
5. An introduction of these bodies from the outside is probably all the more unthinkable, as no other similar substance is otherwise known. Vegetable cellulose is known to exhibit several varieties, but this substance seems to differ from all of them in its lower resistance to reagents, being more strongly attacked by concentrated acids and alkalis than what usually happens with vegetable cellulose.
6. I have looked for them in vain in children so far, so that, like the brain sand, they seem to arise only in later development and may have a potential pathological relevance.

Würzburg, September 4, 1853. Archiv für Pathologische Anatomie, Band IV, Heft 4. (European Journal of Pathology, volume IV, Issue 4).

*ADDENDUM*

Since writing the above note, I have repeated and confirmed my investigations.

A new finding emerged, which is that similar bodies also occur in the higher sensory nerves. I found them most abundantly in the soft, gray intermediate substance of the olfactory nerve, less frequently in the acousticus although the reports of Meissner indicate a relatively great tendency (Zeitschr. f. rat. Med. Neue Folge. Vol. III. p. 358, 363). They were already described in the opticus by Rokitansky and in the retina they were discovered by Mr. Kölliker, based on verbal communications.

Thus, if I have already mentioned above that the ependyma runs without special boundaries between the nerve elements, now the continuous spread of a similar substance within the upper sensory nerves is shown. If I add a series of pathological experiences, the details of which I reserve for another time, I must conclude that a soft basic mass belonging to the large-scale connective substance penetrates and holds together the nervous elements of the centers and that the ependyma is only the part of it that protrudes freely on the surface through the nerve elements. The statement that the epithelium of the cerebral ventricles sits directly on the nerve elements seems to be based on a confusion about this intermediate substance with the actual nerve substance.

I have not yet succeeded in isolating Corpora Amylacea in large quantities to make them accessible for chemical analysis. Nevertheless, there seems to be no doubt about their cellulose nature. No other substance is known that would cause such a reaction, and although I have examined the most diverse animal tissues, although I have examined in detail the other concentric bodies as far as they have appeared to me recently (e.g., in the thymus, in tumors), nothing similar has been found. Although it is very desirable that direct proof should be made that these bodies do not contain nitrogen, the analogy with vegetable cellulose can be regarded as certain even without this proof.

Thus, the communication of these observations was discussed in the united meeting of the anatomical–physiological and the medical sections of the Natural Scientists' Assembly in Tübingen on the 22nd of this month (*Tageblatt der 30sten Versammlung Deutscher Naturforscher und Aerzte. No. 6, p. 62*)^5^ without any reservations. Würzburg, September 25, 1853.

## Comment

In the description made by Virchow, he found these brain aggregates after applying on brain tissue a iodine staining typically used to detect cellulose and other polyglucosans. Indeed, the iodine staining, with specific variations depending on the final aim of the technique, is still used nowadays to detect starch granules and, depending on the color outcome, to differentiate the length and branching of the polyglucosan chains present in specimens (Sakai et al. 1969; Brewer et al. 2020). After the observations made with the limited molecular analysis available in the years of his description and based on the discoveries that the human body could produce sugar in the liver (Bernard 1851), Virchow assumed that CA had a cellulose nature.

Nowadays, the general consensus on CA composition is that their scaffold is made up of polymerized hexoses resembling starch, being glucose the most abundant sugar in these bodies (Sakai et al. 1969). Virchow found these bodies to be mainly associated with the epithelium of the ventricles, the septum, the fornix and some other structures, but not in all the regions of the brain. This allows to assume that his description was made about CA indeed and he was not mistaken for Lafora bodies, which were described several decades later (Lafora and Glueck 1911). In addition, he did not find CA in children, and further studies have described that the presence of these bodies increases with age (Ellis 1920; Mrak et al. 1997). On the other hand, Virchow also tried to find CA in rabbits, but he was unsuccessful. Provided that aging is one of the main factors for CA to accumulate in the brain, it could be assumed that the rabbits used in his investigations would not have been old enough to contain CA in their brains. Indeed, similar structures to CA, named PAS granules, do appear in mouse strains such as B6, AKR or ICR-CD1, but only at old ages such as 7, 12, or 15 months of age, respectively (Jucker et al. 1994; del Valle et al. 2010; Manich et al. 2016). In this sense, the senescence-accelerated mouse prone 8 (SAMP8) mice, a strain with accelerated senescence, begin to show some PAS granules as early as at 3 months of age and the amount of these granules increases with age (del Valle et al. 2010), confirming the close link between CA and aging.

Concerning their origin and composition, it has been suggested that the induction of heme-oxygenase 1 in intracellular oxidative stress would lead to a transformation of mitochondria into CA within different autophagic processes (Schipper and Cissé 1995; Schipper et al. 2019). Other authors point to an external origin (Pisa et al. 2016), and indeed the consideration that CA is a successful protection of foreign material has still not been ruled out. However, the most accepted hypothesis points to an astrocytic origin (Alder 1953; Schipper and Cissé 1995; Augé et al. 2019; Riba et al. 2021b), and the various components that can be found within these bodies and the physiological significance of CA have been a matter of debate in recent years (Augé et al. 2017, 2018; Riba et al. 2021b). The polyglucosan body of CA is actively created in astrocytes, as suggested by the presence of glycogen synthase enzyme in these bodies (Augé et al. 2018). As Virchow pointed out, CA appear concentric, and indeed different components have been described in the periphery or the central part of the granules (Augé et al. 2018), confirming the layered or progressive growth of these structures. The same glucidic structure that build up the scaffold of CA can contain NE recognized by natural IgM antibodies (Riba et al. 2021a). In this sense, natural antibodies can be generated even before birth and are useful to remove waste components or residual cell remnants without inflammatory responses. However, CA remain as intracellular bodies and exit astrocytes only to be released to the CSF (Riba et al. 2019), so neither microglia nor IgMs, which cannot access brain tissue due to the brain–blood barrier, trigger an immune response within the brain neuropil (Riba et al. 2019, 2022a). Indeed, one of the routes that CA take from the brain to the CSF are the periventricular regions, where Virchow already found them and where they accumulate with age (Schipper and Cissé 1995), being the subpial area and the perivenous spaces (through the Virchow–Robin space) other possible exit doors that CA follow from the brain to the CSF. Then, once CA reach the CSF, CA can enter the lymphatic circulation through the meningeal lymphatic vessels and end up in the cervical lymph nodes (Riba et al. 2019). Both in the brain interfaces (as the borders of the ventricles) or already in the lymph nodes, the immune response includes M2 macrophages that recognize CA through CD206 receptors, triggering a non-inflammatory response without inducing tissue damage (Riba et al. 2022a).

However, while no lipid content has been described in brain CA (Alder 1953), several groups have found different proteinaceous substances derived from neurodegeneration, such as tau protein, amyloid-β peptide, S100, or parkin (Augé et al. 2018; Wander et al. 2022; Riba et al. 2023). Other studies have also found bacterial or fungal components in the brain CA of patients with AD, Hungtington’s disease, Parkinson’s disease or even amyotrophic lateral sclerosis (Pisa et al. 2016, 2018; Carrasco et al. 2020). In these brains, the fungal or bacterial components would represent the remnants of previous infections that had been scavenged within CA. In fact, the presence of waste substances from the brain microenvironment in CA has been suggested by different authors (Sbarbati et al. 1996; Cavanagh 1999; Augé et al. 2017; Carrasco et al. 2020). However, it should be taken into account that CA contain NE that can be recognized by IgMs, and considering that contaminant IgMs are often found as contaminants in commercial or custom-made antibodies, several reported results may derive from false positives leading to erroneous or inconsistent theories about the specific components that can be found within CA (Manich et al. 2016; Augé et al. 2017, 2018). Thus, due to these false stainings, various elements of the CA have had or are yet to be reviewed to confirm or deny which substances are indeed entrapped within the scaffold of CA.

Provided that CA amass waste substances, and taking into account the fact that CA themselves can trigger a systematic immune response only when they have exited the brain to the CSF, it is plausible to think that the bodies that Virchow described as cellulose-like accumulations are indeed an orchestrated homeostatic system of brain waste management. In this sense, the term wasteosomes has been recently suggested to name these structures instead of CA, emphasizing the waste products they entrap and to avoid misleading interpretations due to their starch-like amyloid properties and the connotations the term “amyloid” imply in brain pathology (Riba et al. 2021b). However, although described nearly two centuries ago by Virchow, why wasteosomes appear only in aged individuals or in disease remains still a mystery.

A recent paper has suggested that wasteosomes are indeed a hallmark of the insufficiency of the glymphatic system, particularly chronic glymphatic insufficiency (CGI) (Riba et al. 2022b). The glymphatic system enables bulk movement of CSF from the subarachnoid space to Virchow–Robin periarterial spaces. Then, facilitated by aquaporin-4 (AQP4) channels expressed in astrocytic endfeet that ensheathe the brain vasculature, the CSF is filtered and enters the brain parenchyma, mixing with the interstitial fluid (ISF) of the brain (Jessen et al. 2015). Subsequently, due to this brain fluid movement, ISF from the neuropil flows across the brain parenchyma sweeping different solutes and waste products to perivenous spaces, the roots of spinal and cranial nerves and the ependyma that surrounds the cerebral ventricles (Rasmussen et al. 2022), which constitute different brain egress sites. Ultimately, the ISF of the brain and the substances that it contains drain into the dorsal and ventral meningeal lymphatic vessels that surround the brain or into the lymphatic vessels of the soft tissues that surround the skull (Bohr et al. 2022), to finally reach the cervical lymph nodes (Iliff et al. 2012) or even more distal targets. The glymphatic clearance system maintains homeostasis in the brain but, if this cleansing system is disrupted and a glymphatic insufficiency occurs, the result will be the accumulation of waste substances in the brain parenchyma and thus an increase in the number of wasteosomes (Riba et al. 2022b).

However, it should be noted that most of the research on the glymphatic system has been conducted in rodent models. In fact, the basic principles of glymphatic transport in the human brain are not yet fully understood and extrapolating findings from rodent studies to humans should be done cautiously. There may be limitations in directly translating findings from rodents to humans due to differences in brain anatomy, physiology, and overall complexity of the glymphatic system in humans (Benveniste et al. 2019). Specifically, differences between mice and humans have been reported regarding the AQP4 distribution pattern, which also supports the divergences between species in the glymphatic system function (Eidsvaag et al. 2017). Due to these differences, some studies have focused on humans using magnetic resonance imaging (MRI) techniques and positron emission tomography (PET) imaging which have enabled to visualize the flow of CSF in the glymphatic pathways (Eide and Ringstad 2015; Ringstad et al. 2017, 2018) and to study the clearance of waste products through the glymphatic system in the human brain (De Leon et al. 2017; Shokri-Kojori et al. 2018). Yet, the glymphatic system is a newly discovered brain cleansing system (Iliff et al. 2012), the literature on the field is rapidly expanding (Bohr et al. 2022) and the relationship between CA and the glymphatic system is still a matter under study (Riba et al. 2022b).

Virchow found corpora amylacea in areas adjacent to the ventricles (Virchow 1854) and was unable to find them in the cortex or, as he describes, within the interior of the brain substance. However, most recent data describes that these wasteosomes (or corpora amylacea as Virchow described them) are mainly found not only in periventricular areas but also in perivascular and subpial regions of the brain (Sakai et al. 1969; Riba et al. 2019; Xu et al. 2021), being some of these the main drain regions of the glymphatic system (Jessen et al. 2015). Assuming that age is one of the main factors that induce the appearance of CA, it is possible that the brain specimens examined by Virchow (the age of the donors is not specified in his works) were from patients not old enough compared to the brains examined in reports of this century, making it very difficult to find these CA in the cortex or in the perivascular or subpial regions.

The glymphatic system is disrupted in aging, sleep alterations, neurodegenerative diseases, and cardiovascular diseases, among others (Kress et al. 2014; Rasmussen et al. 2018; Hauglund et al. 2020). In parallel, wasteosomes are also more present in aging (Virchow 1854), sleep disorders (Xu et al. 2021), different neurodegenerative diseases (Riba et al. 2021b), and cerebral vascular disorders (Riba et al. 2022b). Hence, when the glymphatic system is chronically altered, there is also a parallel increase in wasteosomes. In this sense, the co-occurrence of these two processes in time does not seem to be unrelated. Indeed, although experimental proof that wasteosomes are a direct consequence of CGI is still lacking, the last perspectives of these two factors indicate that if there is a disruption of the glymphatic system, there will be an increase in the amount of waste material that is not eliminated. Thus, it is straightforward to consider that the number of wasteosomes piling these toxics will increase, being the accumulation of wasteosomes a hallmark of CGI (Riba et al. 2022b). Although Virchow was unable to isolate wasteosomes (or corpora amylacea as he named them) for chemical analysis, recent studies have been able to isolate them from *postmortem* intraventricular CSF samples (Riba et al. 2019, 2021a, 2022a). Now it would be interesting to explore whether wasteosomes can also be obtained from lumbar puncture in living people. Considering that wasteosomes enmesh brain waste substances and that these structures are more abundant in several brain pathologies, the study of CA features (number, ultrastructure, localization, or composition), and the study of the possible existence of disease biomarkers within wasteosomes will offer an invaluable opportunity to sample the brain indirectly in living individuals and use these markers as a diagnostic and prognostic tool of several brain conditions.

## Data Availability

No experimental data or materials have been used for this review other than the published and reviewed bibliography.

## Footnotes

1 Original term.

2 Original term.

3 Translator’s note.

4 Translator’s note.

5 Daily Journal of the 30th Meeting of German Natural Scientists and Physicians. No. 6, p. 62.
